# Robotic-Assisted Radical Cystectomy: Single-Center Analysis of Factors Impacting Clavien ≥ II Complications

**DOI:** 10.3390/clinpract14050143

**Published:** 2024-08-31

**Authors:** Maria Chiara Sighinolfi, Tommaso Calcagnile, Enrico Panio, Simone Assumma, Luca Sarchi, Marco Sandri, Emanuela Santangelo, Michele Petix, Mattia Sangalli, Filippo Turri, Giorgio Bozzini, Marcio Covas Moschovas, Vipul Patel, Bernardo Rocco

**Affiliations:** 1 Department of Urology, ASST Santi Paolo e Carlo, 20142 Milan, Italy; 2 Department of Data Methods and Statistics, University of Brescia, 25121 Brescia, Italy; 3 Department of Urology, ASST Lariana, 22100 Como, Italy; 4 Department of Urology, Advent Health Global Robotics Institute, Celebration, FL 34747, USA

**Keywords:** radical cystectomy, neoadjuvant chemotherapy, robotics, complications, readmission rate

## Abstract

Despite the advent of robotics and the decreasing rate of complications after radical cystectomy, several factors are renowned to impair the early outcomes of this procedure. The aim of this paper is to provide a multivariate analysis (MVA) of patient and surgical procedure-related variables likely to affect postoperative course and 30-day complication rate. Fifty-five robotic-assisted radical cystectomies (RARCs) performed at a single center from July 2021 to March 2023 were enrolled. Baseline demographics, comorbidities, and intraoperative and postoperative data were collected. Uni- and multivariate analyses were performed to evaluate the relationship with Clavien ≥ II complications arising within 30 days of surgery. A postoperative Clavien ≥ II complication was evident in 15 patients (28%), whereas Clavien ≥ III occurred only in 5 (9%). At MVA, the only independent predictor of Clavien ≥ II complications was a prior neoadjuvant chemotherapy (OR 5.6; 95% CI 1.22–25.3, *p* = 0.026). Recognized the small sample size, patients who received a prior NAC should deserve special care within the postoperative course.

## 1. Introduction

Bladder cancer (BC) is the seventh most frequently diagnosed cancer in men, with an age-standardized incidence of 20 new cases per 100,000 per year in Europe [1]. BC has a high mortality rate, and up to 85% of patients with muscle-invasive BC (MIBC) will die from the disease within 5 years of diagnosis if left untreated [2]. Radical cystectomy (RC) is the standard treatment for very high-risk non muscle-invasive bladder cancer (NMIBC) and MIBC [3]. RC is known to be one of the most complex surgeries, as it consists of a disruptive phase that includes radical cystectomy with an extensive lymph node dissection and a reconstructive one that restores the urinary tract. Although bladder preservation strategies have called into question the role of surgery [3,4], advances in technology have made surgery as minimally invasive as possible. The role of robotic surgery has steadily increased in recent years as an important player in the treatment of BC. Its advantages, including magnification, 3D visualization, dexterity, and precision, seemingly address the complexity of the procedure. Those benefits, especially the opportunity for advanced suturing tasks, make robotic-assisted radical cystectomy (RARC) easier if compared to the laparoscopic approach [3,5].

Over the past decade, numerous clinical trials have assessed the oncological safety of RARC, finding comparable outcomes to open surgery in terms of lymph node yield, margin rate, progression-free survival (PFS), and cancer-specific survival (CSS) at various time points [6,7,8,9,10,11]. However, these trials did not specifically address the postoperative course; moreover, most series employed an extracorporeal approach for urinary diversion, potentially limiting the benefits associated with a purely minimally invasive approach.

More recently, two randomized clinical trials (RCTs) compared the robotic and open radical cystectomy (ORC) in terms of postoperative course considering only diversions achieved intracorporeally. Expectedly, the robotic approach outperformed the open one in terms of estimated blood loss (EBL), transfusion rate, wound infection, and venous thromboembolism (VTE), together with a faster overall recovery [12,13,14]. 

In these trials, the clinical outcomes of RC were primarily evaluated through comparisons between robotic and open surgical approaches. Regardless of the approach, complications may arise during the postoperative course; risk factors for developing complications after RC are different and may include age, comorbidities, and type of urinary diversion. Additionally, prior radiation treatment and neoadjuvant chemotherapy (NAC) have also been reported as contributing factors [15].

As a complex intervention, radical cystectomy carries the risk of complications that necessitate invasive management, often classified as Clavien Grade III. These complications are often associated with inherent drawbacks of the surgery itself, including urinary leakage, lymphocele, or disruption of the bowel anastomosis.

These complications are likely to arise from the reconstructive step or from the extensive dissection of a locally advanced disease. On the other hand, medical conditions requiring pharmacological treatment are frequent as well after RC and are defined as Clavien II group, encompassing the need for blood transfusions too. Robotic surgery is expected to decrease the rate of Clavien II complications, given the reduced occurrence of blood loss, wound infection, and VTE [12,13,14].

The aim of the present study is to evaluate risk factors that impact both medical and surgical complications of robotic radical cystectomy, defined as Clavien ≥ II complications, irrespective of the diversion type and the purpose of surgery (oncological and non-oncological).

## 2. Materials and Methods

This study is a retrospective, single-center cohort analysis of patients who underwent robotic-assisted radical cystectomy (RARC) between July 2021 and March 2023. It was conducted at a tertiary care institution in Italy, ASST Santi Paolo and Carlo, Milan. The institution is academically affiliated and serves as a referral and training center for robotic surgery. It is equipped with various robotic systems, including Da Vinci, Hugo RAS, and Versius CMR, facilitating a comprehensive range of surgical interventions.

### 2.1. Study Population

Patients aged 18 years and older were considered for this study. The indication of radical cystectomy for oncologic purposes fitted the European Urological Guidelines for muscle-invasive and non muscle-invasive bladder cancer. In case of NMIBC, the risk calculation found that very high-risk patients can take advantage of an early surgical approach. Cases were evaluated in a multidisciplinary setting and neoadjuvant chemo was performed when appropriate according to guidelines and oncologists’ evaluation. In case of the non-oncologic purpose, the complexity of symptoms and patient’s requirements were considered.

In conclusion, inclusion criteria were the following:

- histologically diagnosed MIBC or very high-risk NMIBC with an indication for radical cystectomy according to EAU Guidelines;
- indication for RC due to post-radiation cystitis.

Cases were enrolled regardless of the type of urinary diversion employed, which could include ureterostomy, intracorporeal neobladder, or ileal conduit.

All patients provided informed consent for the procedure and received extensive counseling about the procedure itself and the options for urinary diversion. Female patients were offered a consultation with a gynecologist to discuss the possibility of organ-sparing procedures. Similarly, male patients were counseled regarding the option of nerve-sparing RARC when oncologically feasible. Surgeries were performed by two robotic surgeons with a high level of expertise.

Data were retrieved from a prospectively maintained database completed by physicians not directly, or only marginally, involved in the surgical team. The variables collected within the database are the following:

- *Baseline:* age, BMI, previous surgery, comorbidities, ECOG, ASA score, Charlson Comorbidity Index (CCI, calculated from https://www.mdcalc.com/calc/3917/charlson-comorbidity-index-cci, accessed on 29 October 2023), clinical stage (cT), clinical nodal status (cN), use of NAC prior to surgery, prior radiation treatment for other disease.
- *Intraoperative data*: complication rate, EBL; any change from pre-operative planning.
- *Postoperative data*: histological type and differentiation of BC; pT/ypT, pN/ypN; histological grade. Surgical margin status, incidental diagnosis of prostate cancer. Length of stay (LOS); complications collected separately and classified according to Clavien–Dindo grading system [16].

Complications are ranked according to the Clavien–Dindo classification, which involves the therapy used to correct each specific adverse event. The Clavien–Dindo focuses on the therapeutic consequences of complications and thus correlates with the complexity of surgery fitting a composite procedure such as RC; it is a simple, reproducible, logical, useful, and comprehensive tool [16]. Its reproducibility was tested in a large cohort and, since then, it has been extensively used in several surgical fields [16].

### 2.2. Perioperative Work-Up 

No bowel preparation was performed prior to surgery. An ERAS protocol was attempted when deemed feasible [11]. However, early mobilization starting from the 1st to 2nd postoperative day was encouraged in all patients, together with oral feeding. A treatment with low molecular weight heparin was performed for four weeks according to guidelines. In case of a neobladder or ileal conduit, ureteral catheters were removed on postoperative days 8 to 14; in case of a neobladder, the trans-urethral catheter was removed on postoperative days 16–21. Complications were graded using the Clavien–Dindo classification (CDC) [16]. Patients were re-assessed weekly up to 30 days after discharge.

### 2.3. Surgical Technique

The patient is placed in an 18° Trendelenburg position.

After performing the docking of the robotic system, ureters are bilaterally identified and isolated from the above iliac vessels until bladder insertion. The ureters are then closed. In males, the peritoneum is incised at the seminal vesicle level, and a plane is developed between Denonvillier’s fascia and the posterior face of the prostate (between the bladder and vagina in females). Lateral aspects of the bladder are developed bilaterally, and vesical pedicles are transected after being clipped. Lifting the bladder with the fourth arm—which should be extensively and properly used, especially in challenging cases with high tumoral burden—facilitates the access to the pedicles. If considered appropriate, preservation of the neurovascular bundles is performed in males. Then, an inverse U peritonectomy is carried out and the anterior face of the bladder is developed, and the Santorini complex is excised and sutured. The urethra is isolated and then incised after a large Hem-o-lok is placed to prevent urine spillage. Frozen sections of distal ureters and urethra are carried out.

In women, the standard procedure includes the removal of the bladder, the entire urethra, the anterior vaginal face, the uterus, the distal ureters, and the pelvic lymph nodes; in case of neobladder reconstruction, a pelvic organ-preserving strategy may be pursued, preserving the uterus and vagina to support the reservoir. Previous gynecological counseling is mandatory to evaluate sexual function, gynecological history, and possible prolapses.

The extended LND includes the removal of all the standard pelvic lymph nodes, the ones located in the region of the aortic bifurcation, presacrally, those close to the common iliac vessels (medially to the crossing ureters), those close to the lateral borders of the genitofemoral nerves, those located caudally of the circumflex iliac vein and the lacunar ligament, and the node of the Cloquet. In the present series, a single case required a super-extended LND due to the extension of macroscopically pathological nodes cranially reaching the level of the inferior mesenteric artery.

In the case of neobladder reconstruction, the technique described by Asimakopoulos et al. [17] is chosen. A 40–50 cm ileal segment is isolated; the portion with a more adequate mesenteric length is elected to be brought down to the pelvis. The median part of the isolated ileal segment is pushed towards the urethral stump, and the ileo-urethral approximation is accomplished as the first step. A modified posterior reconstruction is performed before the anastomosis. Then, both ileal segments are isolated at each side using a mechanical stapler, and an ileal–ileal anastomosis is accomplished. The reverse tubular U-segment of the ileum is detubularized to configure the neobladder. The reconstruction starts from the suture of the posterior plane, and then the cranial part is folded downwards toward the bladder neck to create the orthotopic reservoir with two lateral limbs. Finally, a uretero–neobladder anastomosis is performed with direct anastomosis of each spatulated ureter in the dorsal part of the limbs (monocryl 4–0).

### 2.4. Endpoint

The primary endpoint is to evaluate factors related to Clavien ≥ II complications occurring up to 30 days after discharge. Among Clavien II complications, blood loss and transfusion requirement, fever, pneumonia, urinary tract infection, and lymphocele requiring antibiotics were included.

### 2.5. Statistical Analysis 

Continuous variables were summarized as means ± standard deviations, while categorical variables were described as absolute and relative frequencies. To investigate the associations between the binary outcome of Clavien ≥ II complications and potential predictors, logistic regression analysis was utilized. This model allowed us to estimate the odds ratios (with 95% confidence intervals), identifying how strongly each predictor is associated with the likelihood of experiencing Clavien ≥ II complications.

Additionally, the associations were further examined using the 4-category Clavien–Dindo variable through ordinal logistic regression analysis. This method enabled the evaluation and quantification of the relationship between each variable and the graded severity of complications as defined by the Clavien–Dindo scale.

Lastly, we also utilized the multivariable versions of the aforementioned models to estimate the association between outcomes and predictors, controlling for the effect of a set of adjustment variables.

All analyses were performed using STATA 16 (StataCorp. 2019. College Station, TX, USA). A *p*-value of less than 0.05 was considered statistically significant for all tests.

## 3. Results

Overall, 54 RARC procedures performed between July 2021 and March 2023 were enrolled, with a cohort of 45 males and 9 females. The mean age of participants was 68 years (range: 46–86); the mean BMI was 26 (range: 17–36). Of the patients included, 51 were diagnosed with bladder cancer, whereas 3 underwent RARC for post-radiation cystitis. The median CCI was 4 (IQR 4–5; range 2–9), with ten patients having received prior neoadjuvant chemotherapy (NAC). Except for two patients who underwent RARC with the Hugo RAS system, all cases were performed using the Da Vinci Xi system. Regarding urinary diversion, 22 patients received an orthotopic neobladder, 8 had an ileal conduit, and the remaining cases had a ureterostomy. Postoperatively, 15 patients (28%) experienced complications classified as Clavien–Dindo Grade II, while 5 (9%) had complications of Grade III. In Table 1, the demographic and clinical characteristics of the entire study cohort are summarized. In Table 2, these characteristics are compared between patients with Clavien 0–I postoperative complications and those with Clavien II–III complications. Pathologic staging pointed out 14 cases of high-grade non muscle-invasive BC, 13 cases of ≥T3, and the remaining were pT2 cases. The four patients who underwent surgery for non-oncological purposes did not have BC at final pathology.

Using logistic regression, we assessed the discriminatory power of various potential predictors (age, gender, BMI, NAC, CCI, prior abdominal surgery, console time, type of diversion, and bowel use) for Clavien ≥ II postoperative complications (Table 2). The analysis revealed that only NAC was significantly associated with Clavien ≥ II complications, evidenced by an odds ratio (OR) of 5.6 (95% CI 1.22–25.3, *p* = 0.026).

Considering the Clavien–Dindo classification as an ordinal categorical variable with four categories (from Grade 0 to Grade III) and applying an ordinal logistic model, the predictive capacity of NAC was confirmed, evidenced by an OR of 6.1 (95% CI 1.36–27.5, *p* = 0.018). The percentages of the patients receiving NAC within the groups categorized by Clavien grades 0, I, II, and III were 8%, 11.1%, 26.7%, and 60%, respectively. The above results were also confirmed by a multivariable analysis (Table 2). Considering Clavien ≥ II postoperative complications and a multivariable logistic model with sex, age, and BMI as adjustment variables, NAC showed an OR of 12.5 (95% CI 2.2–70.4, *p* = 0.004). Meanwhile, considering NAC as an ordinal categorical variable, the adjusted OR was 14.5 (95% CI 2.84–73.9, *p* = 0.001).

## 4. Discussion

Radical cystectomy is one of the trickiest and most invasive surgical procedures in urology. Between 19% and 99% of perioperative complications are reported to occur during the first 90 days following surgery; complications requiring an intervention under anesthesia (Clavien ≥ III b) may occur in 18% of patients.

Robotic surgery may improve optical visualization and surgical maneuvering for retraction, exposure, and tissue resection; however, the complexity and length of RC is still responsible of a considerable number of complications. Beyond threatening adverse events linked to the complexity of surgery, mostly graded as Clavien III, the burden of radical cystectomy is also based on medical complications requiring a therapy, that are included in the Clavien II group. The latter may be debilitating as well and may have an impact on length of stay (LOS) and time to recovery. Furthermore, it should be remarked that complications requiring medical treatment may occur within a patient cohort that is often frail, old, and already hindered by the oncological disease. 

The current series aims to evaluate all factors likely to impact the development of a Clavien ≥ II complication. The use of NAC prior to surgery turned out to be the only independent factor adversely impacting the 30-day complication rate under a multivariate analysis.

We included in the analysis other renowned factors related to the patient, such as age, BMI, and CCI; these variables are recognized to impair the outcomes of RC, irrespectively of the surgical approach. A high BMI—in particular ≥ 25 kg/m^2^—was found to be a strong predictor of both 30-day and 90-day complications [18,19]. In previous studies, the Charlson comorbidity index has been invoked as a predictor of the occurrence of grade Clavien ≥ III complications [20]; similarly, a higher CCI score was found to be associated with 90-day readmission (OR 1.23) [15]. In the current series, age, BMI, and CCI were found not to be linked to the development of Clavien ≥ II complications at MVA. Similarly, gender turned out not to be related to complication occurrence. This is in contrast with previous findings that showed that females may experience more complications, worse survival outcomes, and more advanced diseases [21].

Another factor we considered was the diversion complexity. According to the EAU Guidelines, most complications are diversion-related [22,23]. It is recognized that the ureteral diversion to the abdominal wall is the simplest form, as operating time, complication rate, stay at intensive care, and LOS are lower in patients treated with ureterostomy [22]. The decision to utilize or not the bowel to perform a urinary diversion, especially the ileum, significantly impacts the likelihood of complications, since it adds the intestinal steps and increases the invasiveness of the procedure. According to Deliveliotis et al., patients who undergo RC with an ileal conduit have a longer operative time and more postoperative complications, compared to patients who undergo ureterocutaneostomy [24]. The overall morbidity of RC may be increased by the use and manipulation of the ileum for the urinary reconstruction. In the current series, the use of the bowel and the diversion choice were not independent predictors of Clavien ≥ II complications. This is peculiar, considering that a large number of cases were managed with neobladder reconstruction, the most complex surgical diversion; furthermore, all diversions were reconstructed intracorporeally, and this has been previously considered another risk factor for complications as well [15].

Within the current series, a neoadjuvant chemotherapy based on cisplatin was the only independent predictor of Clavien ≥ II complications. Cisplatin-based NAC has been used since the 1980s to improve survival in patients with cN0M0 disease. Muscle-invasive bladder cancer is an aggressive tumor that can spread micro-metastases at diagnosis. Thus, NAC provides an opportunity to eradicate the micro-metastatic location and it has been proven to increase 5-year overall survival by 5–7%. Patients who respond to NAC usually have a more favorable pathological response, defined as attainment of ypT0N0/ypTisN0 at RC [22]. From the phase III RCT of Griffiths, at a median follow up of eight years, NAC provided a 16% reduction in mortality risk and a benefit regarding distant metastasis [25]. More modern chemotherapeutic regimens merge gemcitabine and cisplatin [22]. 

NAC eligibility and elective factors have been argued over time. In a recent single institution study including MIBC patients from 2005 to 2021, Patel et al. addressed the topic on 380 MIBC patients, 40.5% of whom received NAC [26]. The authors found that, beyond contra-indications, factors for which patients do not receive NAC were patient symptoms (7.8%), disease progression concern (7.0%), patient preference/refusal (20.3%), and provider discretion (8.1%). Nevertheless, NAC utilization increased over time, from 34.2% in the early years up to 85.7% on eligible patients in 2016–2021. Renal dysfunction (16.6%), clinical contra-indications (4.7%), salvage setting (2.1%), and histology (5.3%; total N = 109) were the reasons for non-eligibility [26]. Despite its growing use, the adherence to NAC is still suboptimal, and a high inter-hospital variation is evident: within the nationwide Netherlands Cancer Registry, only 26% of cT2 receive NAC, with a discrepancy between institutions ranging from 7% to 57% [27]. 

The oncological advantage provided by NAC has also been confirmed on data from the International Robotic Cystectomy Consortium [28]: within this database consisting of 1370 patients, 353 of whom (26%) received NAC before robotic RC, NAC patients had higher rates of survival, pathological downstaging, and margin-negative resections, together with fewer distant recurrences [28].

Overall, NAC did not seem to affect the morbidity of surgery, as clearly stated in the EAU Guidelines. The RCT from Grossmann et al., published in 2003 and quoted within the EAU Guidelines, showed the same distribution of grade 3–4 postoperative complications in patients with and without prior NAC [29]. In the combined Nordic trials (N = 620) published in 2004, NAC did not provide major adverse effects impacting the rate of performable cystectomies. The cystectomy frequency was 86% in the experimental arm and 87% in the control arm, with 71% of patients receiving all three chemotherapy cycles [30].

Other retrospective large series confirmed these results thereafter. 

In 2014, Johnson et al. [31] performed a review of the American College of Surgeons National Surgical Quality Improvement Program (NSQIP) database to identify RC patients from 2005 to 2011. On multivariable logistic regression, NAC was not a predictor of complications, reoperation, wound infection, and wound dehiscence. 

In the same year, Gandaglia et al. [32] reviewed data from the Surveillance Epidemiology and End Results Medicare-linked database to compare outcomes of RC with and without prior NAC. They found no differences in rate of complications, hemoglobin drop, prolonged LOS, readmission, and mortality. 

In 2019, Milenkovic et al. [33] compared the complication rate of 102 patients receiving NAC prior to cystectomy to those of 389 patients not receiving NAC, after score covariate adjustment. Despite a higher number of hematologic adverse events after NAC, with a higher transfusion rate, the overall number of complications was similar between groups and at 30 days.

To note, these series did not consider the surgical approach to RC, and most of the surgeries were supposed to be performed with an open approach.

In the current robotic series, NAC was the only factor independently linked to a Clavien ≥ II complication. To note, this group included either a medical condition requiring treatment or a surgical complication needing an invasive management. Given that drawbacks deriving from surgery accounted only for 4 out of 54 patients, it can be speculated that the major impact of NAC occurs on the Clavien II complication rate.

These outcomes are consistent with the prior robotic series reported by Johar et al. in 2013 [34]. 

The preliminary report from the International Robotic Cystectomy Consortium (IRCC) database of 939 RARC patients described that NAC was an independent predictor of any complications, including high-grade complications.

However, an update from the same group (IRCC) reported in 2020 focused on 1156 MIBC-alone patients who underwent RARC [15,35]. The authors compared patients with NAC receipt (298, 26%) with those without. In the updated series, NAC was not significantly associated with operative time, LOS, 90-day complications, reoperation, and mortality; however, it was associated with 90-day readmission. Anemia due to NAC was considered responsible for 40% of hematological toxicities and a desmoplastic tissue reaction of NAC was invoked as the cause of a higher 90-day readmission rate. The authors suggested pre-operative counseling about a higher readmission rate on patients with NAC receipt. 

Of note, the high-grade complication rate was firmly comparable between NAC and non-NAC patients. However, a trend towards an increased overall complication rate at 90 days was evident. Actually, the IRCC database described increased NAC use across decades, ranging from 10% of cases in 2006 to 2007 to 42% in 2016 to 2017; together with the occurrence, overall complications also significantly increased from 29% of cases in 2006 to 2007 to 64% in 2016 to 2017 (*p* < 0.001). Whereas the incidence of 90-day high-grade complications remained stable across time (12% in 2006 to 2007 vs. 13% in 2016 to 2017), a trend towards a higher number of low-grade complications could be hypothesized. NAC use could be responsible for some conditions requiring medical treatment during a postoperative course of RARC. In our series, the inclusion of Clavien II complications registered conditions such as blood loss, urinary infection, and pneumonia; accounting for these adverse events would have been responsible for the impact of NAC on the 30-day complication rate in our series. 

Remarkably, when considering long-term data, an adverse impact of NAC could be exerted on renal function according to a recent study from Ho et al. [36]. The authors compared 234 patients with MIBC and NAC receipt to a parallel cohort who did not undergo NAC. One year after surgery, a 17% decline in eGFR was evident in NAC patients together with a nearly doubled incidence of stage ≥ 3 CKD compared to non-NAC patients. The authors concluded that patients who underwent RC without NAC were experiencing less renal function loss at one year; furthermore, they discussed that the use of perioperative chemotherapy on large databases had been evaluated as a multivariable analysis, and thus complications such has eGFR decline would have not been captured and would have remained underreported. 

The current series presents several limitations. First, the small sample size and the single-center fashion preclude any conclusion. Even if from the current series NAC could have an impact on the complication rate, we strongly remark on its strong impact on oncological endpoints that should drive clinical decisions. Second, as an observational and retrospective investigation, our result should be interpreted with caution as the study was not powered to address differences between NAC and non-NAC patients; furthermore, we did not account for dose adaptations in NAC regimens. Third, we failed to address intraoperative complications: the impact of pre-operative variables on surgical course could be of interest and should be further evaluated, in robotic and non-robotic surgical settings.

## 5. Conclusions

Despite some limitations, the current series highlights the possibility of an increased rate of Clavien II complications after NAC. However, NAC remains a powerful treatment strategy to improve the oncologic outcomes of BC patients. Improved counseling and deep care of patients who underwent NAC should be advised, to prompt an early diagnosis of adverse events and timely management. Further studies await on the issue.

## Figures and Tables

**Table 1:** Descriptive analysis of the entire cohort; continuous variables are reported as mean and standard deviation while frequencies are reported as number and percentage.

Variable	N = 54
Age	68.2 ± 10.5
Gender	45 males; 9 females
BMI	26.1 ± 3.74
NAC	10/51 (19.6%)
Prior abdominal surgery	9 (20.0%)
CCI	4.8 ± 1.5
Type of diversion	22 neobladder; 8 UICS; 24 UCS

**Table 2:** Demographic and clinical variables for patients with Clavien 0-I and Clavien II-III; continuous variables are reported as mean and standard deviation, while frequencies are reported as number and percentage. For each variable, the odds ratio (OR) along with its 95% confidence interval (95% CI) and the *p*-value (*p*) are also reported.

Variable	Clavien 0–I (N = 34)	Clavien ≥ II (N = 20)	OR (95% CI)	*p*
Age (years)	69.0 ± 10.7	67.2 ± 10.3	0.98 (0.93–1.04)	0.6
Gender (male)	29 (85.3)	16 (80.0)	0.7 (0.13–4.02)	0.9
BMI (kg/m^2^)	26.2 ± 4	25.9 ± 3.5	0.98 (0.83–1.15)	0.8
NAC	3 (8.8)	7 (35.0)	5.6 (1.22–25.3)	0.026
Prior abdominal surgery	5 (18.5)	4 (22.2)	1.25 (0.21–6.98)	1
CCI	4.8 ± 1.6	4.7 ± 1.4	0.94 (0.63–1.37)	0.8
Console Time (min)	355.1 ± 117.5	370.3 ± 129.8	1.00 (0.99–1.01)	0.7
Type of Diversion				
UICS	6 (17.7)	2 (10.0)	0.59 (0.05–4.47)	0.9
Neobladder	14 (41.2)	10 (50.0)	1.24 (0.33–4.86)	0.9
Bowel use	20 (58.8)	12 (60.0)	1.05 (0.30–3.81)	1
Purpose of cystectomy	3 (8.8)	0 (0.0)	0.42 (0.01–4.1)	0.5

## Data Availability

Data will be shared upon appropriate request.
